# Omics Analysis Reveals the Mechanism of Enhanced Recombinant Protein Production Under Simulated Microgravity

**DOI:** 10.3389/fbioe.2020.00030

**Published:** 2020-02-20

**Authors:** Jie Huangfu, Hye Su Kim, Ke Xu, Xiaoyu Ning, Lei Qin, Jun Li, Chun Li

**Affiliations:** ^1^Department of Biochemical Engineering/Institute for Synthetic Biosystems, School of Chemistry and Chemical Engineering, Beijing Institute of Technology, Beijing, China; ^2^China National Research Institute of Food & Fermentation Industries, Beijing, China; ^3^Key Lab for Industrial Biocatalysis, Ministry of Education, Department of Chemical Engineering, Tsinghua University, Beijing, China

**Keywords:** simulated microgravity, omics, recombinant protein, ribosome protein assembly, protein folding

## Abstract

Simulated microgravity (SMG) is regarded as a suitable environment to produce recombinant proteins. This study showed that β-glucuronidase expressing *Escherichia coli* had higher productivity of recombinant protein and higher plasmid copy number under SMG compared with the normal gravity condition. The cellular changes were analyzed at both transcriptomic and proteomic levels. The upregulation of a group of ribosome/RNA polymerase genes and a cluster of genes involving energy metabolism at transcriptomic level stood out for improved production of recombinant protein under SMG. The protein folding modulators such as chaperones were upregulated at proteomic level, which could be a result of the increased activity of protein synthesis and can help recombinant protein production. Protein export was also strengthened, which was revealed at both transcriptomic and proteomic levels. The results demonstrated that SMG is a favorable environment for recombinant protein production arousing the upregulation of protein synthesis, protein folding, and protein export.

## Introduction

Microgravity is a special environmental condition for microorganisms. The significant characteristics of this extreme and unique environment are the low sedimentation, low shear stress, and low turbulence (Nickerson et al., 2004). The reduced gravity might elicit a number of distinct physiological variations to microorganisms, such as microbial growth (Rosenzweig et al., 2010), resistance to multiple stresses and antibiotics (Gao et al., 2001; Wilson et al., 2002), and substrate utilizations (Brown et al., 2002). Since the microgravity experiment in space takes enormous resources and time, the techniques of clino-rotation have been devised to simulate microgravity on the ground. Under the simulated microgravity (SMG), microorganism has a shorter lag phase, a higher growth rate, and a higher cell density compared to the normal gravity (NG) (Baker et al., 2004).

*Escherichia coli* is widely used for expressing recombinant proteins; however, there are still bottlenecks for obtaining large amounts of soluble and functional proteins (San-Miguel et al., 2013). Variation in environmental condition was reported to influence the recombinant protein production (Hoffmann and Rinas, 2004; Jamal et al., 2009). Some studies have demonstrated that SMG had impact on the heterologous protein production. It was reported that SMG enhanced the production of recombinant proteins of LacZ and glycodelin in the human cells compared with a stirred bioreactor under NG (Navran, 2007). A previous study found that SMG enhanced the expression of the recombinant β-glucuronidase in *E. coli* (Xiang et al., 2010). However, the research about the mechanism of SMG on the expression of recombinant proteins by bacteria is still lacking.

The potent expression of desired recombinant proteins involves efficient protein translation and functional protein folding. The ribosomes, consisting of a huge complex RNA and proteins, are protein factories for protein synthesis and assembly. The ribosome is comprised of two subunits: large subunit [5 small ribosomal RNA (rRNA), 23 small rRNA, and 33 r-proteins] and small subunit (16 small ribosomal RNA and 21 r-proteins) in *E. coli* (Kaltschmidt and Wittmann, 1970). Ribosomal proteins have significant function on maintaining the rRNA structure and messenger RNA (mRNA) helicase activity in ribosome biogenesis (Ogle et al., 2001; Takyar et al., 2005). For protein folding, the nascent polypeptide chains have received assistance from many molecular chaperones (Frydman and Hartl, 1996). In *E. coli*, different proteins interact with different chaperones according to polypeptide chain length. Small proteins (<30 kDa), taking 70% of total, interact with Trigger factor tig, a ribosome-associated chaperone (Hartl and Hayer-Hartl, 2009). Longer proteins, belonging to 20% of total, interact with dnaK and dnaJ (Hsp70 system) (Clerico et al., 2015). About 10% of polypeptide chains are transported to groEL and groES chaperonin system (Hayer-Hartl et al., 2016). Understanding how SMG affects protein translation and protein folding could be profitable to discover new potentials for increased recombinant proteins.

In this study, we examined the effects of SMG on expressing *Aspergillus oryzae* β-glucuronidase (pGUS) by the recombinant *E. coli*. The potent changes of ribosome protein assembly and protein folding were revealed by the multilevel omics analysis. This result could be helpful to comprehensively understand the physiological adaptation of recombinant *E. coli* under SMG and provide new insight into developing unconventional bioprocess to enhance recombinant protein production.

## Materials and Methods

### Strain and SMG Cultivation

The recombinant *E. coli* BL21 (DE3)/pET28a-pGUS previously constructed (Shi et al., 2011) was authorized and used in this study. Conditions referred as SMG and NG were designed by rotating the high-aspect rotating-wall vessel (HARV; diameter, 8 cm; depth, 1 cm) horizontally and vertically on the rotating cell culture systems (RCCS-4D, 50 ml; Synthecon Inc., Houston, TX), respectively.

An overnight bacterial culture was inoculated into 30 ml Luria–Bertani (LB) medium (10 g/L tryptone, 5 g/L yeast extract, and 10 g/L NaCl) in a shaker flask at 37°C for 10 h. The cell suspension was diluted (1:10) in two HARV vessels filled with fresh LB medium containing 50 μg/ml kanamycin. Both of the two HARVs were first incubated at 37°C for 4 h. After that, the cells were induced by adding 0.8 mM isopropyl β-d-1-thiogalactopyranoside (IPTG). The SMG culture process was carried out under different rotary speeds (10, 15, 20, and 30 rpm), induction temperatures (17, 27, and 37°C), and induction time (4, 6, and 8 h) to find the optimal condition for the efficient recombinant protein production. The NG culture process was carried out at the same condition. Cell growth curve was tested periodically by measuring the OD600 using an ultraviolet spectrophotometer (Hitachi, Japan) through triplicate independent experiments. All experiments were carried out in triplicate.

### Protein Expression Analysis and Enzyme Assay

The strain after cultivation was collected by centrifugation at 8,000 rpm for 15 min. The pellets were suspended in 200 mM phosphate buffer (pH 6.0) and ultrasonicated on the ice. After centrifugation, the supernatant (soluble protein) and pellet (inclusion body) were separated. A semiquantitative determination of the soluble protein and inclusion body was detected by sodium dodecyl sulfate polyacrylamide gel electrophoresis. Bovine serum albumin was used as an internal standard to determine the total protein concentration by the Coomassie brilliant blue R250 staining method. The pGUS activity was assayed by high-performance liquid chromatography (Shimadzu, Japan) from cell crude extract after sonication using glycyrrhizin as the substrate. One unit of activity was defined as the amount of enzyme that released 1 μmol of biosynthesized β-d-mono-glucuronide-glycyrrhizin in the reaction mixture per minute (Feng et al., 2006). All the experiments from a biological sample were carried out in triplicate.

### Plasmid Stability and Copy Number Analysis

The strains were cultured overnight in liquid LB medium without kanamycin under SMG and NG, respectively. Then, the cells were collected and diluted (1:100) into fresh liquid LB medium at every 8 h to continue SMG and NG culturing. Fifty microliters of diluted sample (10^4^ cells/ml) was plated on LB plate (without kanamycin) and incubated at 37°C overnight. After that, the colonies were stamped on selective plates (with kanamycin). The relative ratio of colonies on the plates with kanamycin represents the plasmid stability. Three induction temperatures (17, 27, and 37°C) were chosen to investigate the SMG effect on the plasmid copy number. After adding 0.8 mM IPTG for 4 h, the cells were collected, and the plasmids were extracted. The DNA quantity was assessed using the NanoDrop 2000c spectrophotometer (Thermo Scientific, Waltham, MA). The plasmid number was calculated as the plasmid DNA concentration per OD_600_.

### RNA-seq

Cells under SMG and NG were incubated at 15 rpm and 27°C for 4 h, and subjected to IPTG induction at 17°C for 4 h. After that, total RNA was isolated using the RNA isolation system (Roche). Genomic DNA was removed using DNaseI. RNA quality was assessed using the NanoDrop 2000c spectrophotometer. Sequencing was carried out by Solexa Genome Analyzer commercially. To obtain information regarding the expression level among the genes, the number of relative reads per coding region using a window of 250 bp was calculated. Gene expression was calculated using the transcripts per million (TPM) method. The raw reads of 35 bp were truncated as 28-mers and remapped with the Efficient Local Alignment of Nucleotide Data allowing for 1 and 2 nt mismatches. The output file containing only the sequences that mapped once in the genome was further analyzed to ascertain genome coverage and to assign the number of reads per locus (open reading frame or intercistronic region). To identify the differential expression genes (DEGs), the libraries were initially compared by pairs; for this, the number of reads for each coding region was determined. The number of total reads was normalized between these libraries, and the ratio of reads between SMG and NG was calculated. The genes showing a ratio larger than 2.0 and lower than 0.5 were considered potential candidates. Finally, the number of reads for the four libraries was normalized, and the Student's *t*-test was applied for each gene. Those genes that showed a *P*-value lower or equal to 0.05 (corrected for multiple testing) were considered as statistically significant. The genome sequence and annotation files of *E. coli* K12 MG1655 were obtained from NCBI, and the experimentally verified operons in the bacterium were downloaded from RegulonDB (http://regulondb.ccg.unam.mx/). Categories of differentially expressed genes were identified according to Gene Ontology and Kyoto Encyclopedia of Genes and Genomes using Cytoscape software. On average, 6,415,574 and 6,603,164 reads were obtained from both *E. coli*-pGUS-SMG and *E. coli*-pGUS-NG, respectively. The mapping statistics of the samples were summarized in Table S1. The levels of DEGs were calculated on TPM. All of the genes with a TPM ≥ 0.1 were used as DEGs in the following analysis.

### Proteomic Analysis

Cells were incubated and inducted at the same conditions with previous description for RNA-seq. The cells were then centrifuged and washed with ice-cold phosphate-buffered saline (pH 7.2) for three times. Then, the cells were suspended in an ice-cold lysis buffer containing 8 M urea in 50 mM Tris–HCl (pH = 7.4), 65 mM dithiothreitol, 1 mM ethylenediaminetetraacetic acid, 1% (v/v) Triton, 1 mM phenylmethylsulfonyl fluoride (added freshly), 2% (v/v) protease inhibitor cocktail (added freshly), and phosphatase inhibitor cocktail (1 tablet/10 ml of lysis buffer, added freshly), and ultrasonicated in an ice bath. The supernatant was reserved for the determination of protein content using the bicinchoninic acid method. Proteomic analysis was performed through precipitation by chloroform/methanol treatment, then redissolution in 0.2 ml buffer containing 8 M urea, 50 mM Tris–HCl, pH 8.2. Two milligrams of the above redissolved proteins was reduced by 20 mM dithiothreitol at 37°C for 2 h and oxidized by 40 mM indole-3-acetic acid at 25°C for 45 min in the dark. The protein mixture was further diluted to 2 ml with 50 mM Tris–HCl buffer (pH = 8.3). After adding 40 μg of sequencing trypsin, the protein mixture was digested at 37°C overnight. The obtained digests were reserved at −80°C. The tryptic digests were desalted with C18 solid-phase cartridges and lyophilized. Protein analysis technology was used by LTQ-OrbitrapVelos mass spectrometer (Thermo, San Jose, CA) with one-dimensional reversed-phase liquid chromatography separating system in the positive ion mode. MS/MS experiments of the five most abundant precursor ions were acquired, and the fragmentation data were exported using the Data Analysis Software (version 3.4, Bruker Daltonic). The protein identification was validated by the *E. coli* open reading frame protein database using the MASCOT Daemon (version 2.1.3) search engine. The results were filtered using the SFOER software with optimized criteria, and the corresponding false discovery rate was below 1%. Proteins with a log2(fold change) >1 and log2(fold change) < −1 (*P* < 0.05 for the *t*-test of each of the two samples) were assigned as differential expressed proteins (DEPs).

## Results

### Characteristics of the Recombinant *E. coli*-pGUS Under SMG

**Figure 1:**
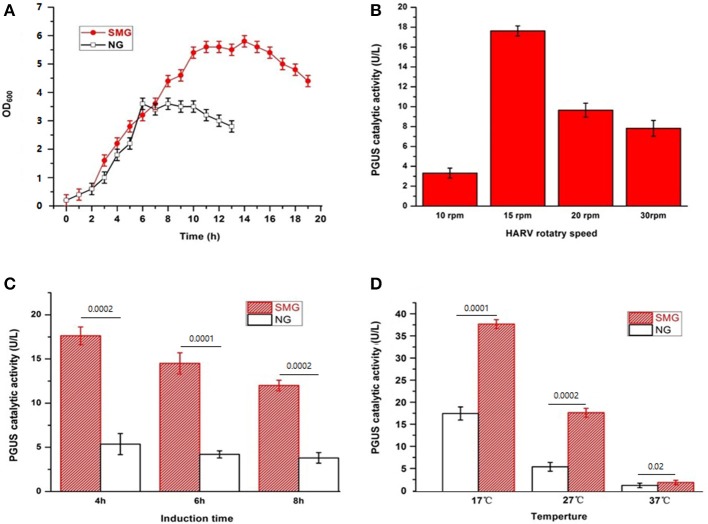
The characteristics of the recombinant *E. coli*-pGUS under SMG and NG. **(A)** Growth curve of the strain at 37°C under SMG and NG at 15 rpm. Optimal HARV rotary speed **(B)**, induction time **(C)**, and induction temperature **(D)** for the recombinant pGUS expressed under SMG were determined. The default condition for the optimization of induction was 15 rpm, 4 h, and 27°C. Significance was assessed using two-sided, paired Student's *t-*test, and *P*-values are indicated as numbers in the graphs, *n* = 3.

Our previous studies have shown the significant improvement of recombinant protein secretion and glycosylation in pGUS expressing *Pichia pastoris* (Huangfu et al., 2016). Now, we want to discover the impact of SMG using *E. coli*-based expression system as the easiest and cheapest host. The growth curves of the strain *E. coli*-pGUS under SMG at 27°C exhibited the enhanced growth rate and the delayed entering into stationary phase (Figure 1A). The strain under SMG at 37°C also showed higher growth rate compared with that under NG (data not shown). The maximum catalytic activity of recombinant pGUS appeared under SMG with 15 rpm, 4 h IPTG induction, and 17°C induction temperature (Figures 1B,C). At different induction temperatures (17, 27, and 37°C), the enzyme activities increased by 2.16-, 2.46-, and 1.55-fold compared with NG, respectively (Figure 1D).

**Figure 2:**
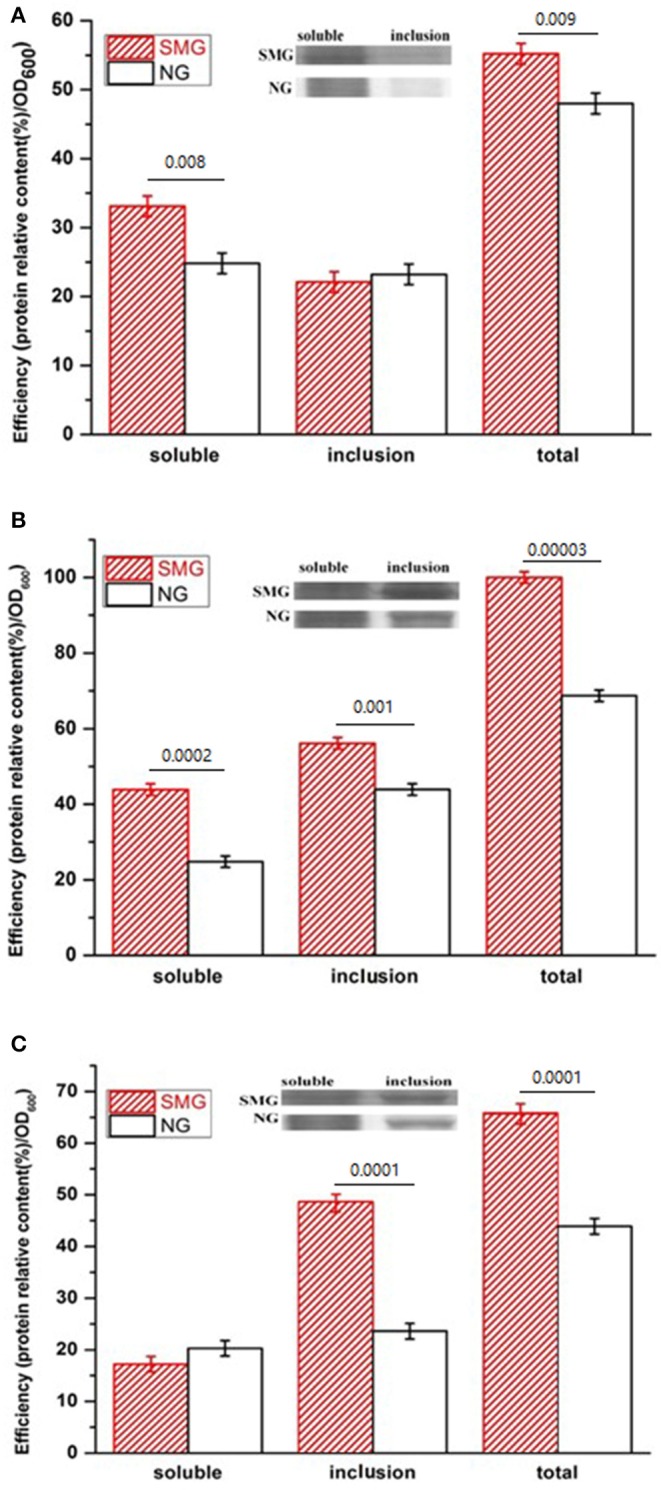
Concentration and activity of the recombinant pGUS expressed under SMG and NG at different temperatures. **(A)** 17°C, **(B)** 27°C, and **(C)** 37°C. Significance was assessed using two-sided, paired Student's *t-*test, and *P*-values are indicated as numbers in the graphs, *n* = 3.

**Figure 3:**
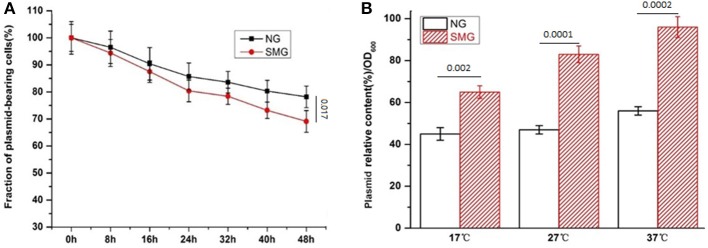
The stability **(A)** and copy number **(B)** of the plasmids in the recombinant *E. coli*-pGUS under SMG. For **(B)**, significance was assessed using two-sided, paired Student's *t-*test, and *P*-values are indicated as numbers in the graphs, *n* = 3.

The pGUS expression efficiencies of the total protein increased under SMG at all inducing temperatures, which increased by 15.3, 48.2, and 52.4% at 17, 27, and 37°C, respectively (Figure 2). SMG also had clear effects on the plasmid stability and the plasmid copy number (Figure 3). The plasmid stability under SMG was slightly lower than that under NG (Figure 3A), but the plasmid copy number under SMG was significantly higher than that under NG (Figure 3B). These results indicated that the cells growth, enzyme activities, and protein expression efficiencies were all facilitated under SMG. Moreover, the higher plasmid copy number under SMG might result in higher protein productivity.

### Transcriptome Revealed the Mechanism of Enhanced Protein Expression Under SMG

**Figure 4:**
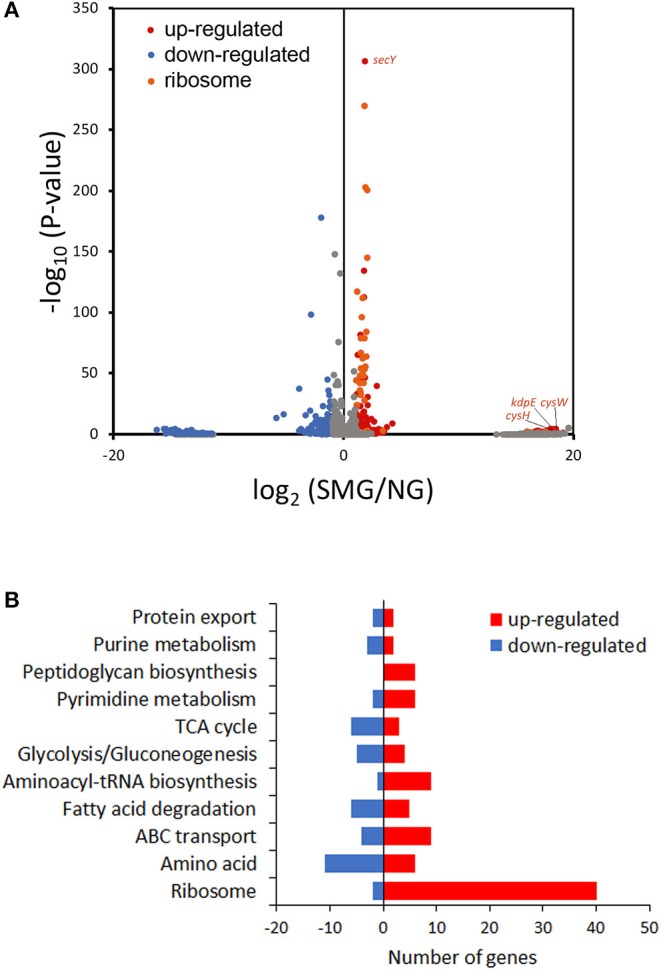
Transcriptome analysis. **(A)** Volcano plot of the transcriptome data. **(B)** DEG numbers in the most differential Kyoto Encyclopedia of Genes and Genomes pathways.

This study compared transcriptomic performance under SMG and NG by RNA-seq (Figure 4A). Among the DEGs between SMG and NG, 316 genes were upregulated and 276 genes were downregulated. The most differential pathways were clustered into the categories of translation hub (ribosomal, RNA polymerase, and aminoacyl-tRNA biosynthesis), metabolism hub [glycolysis/gluconeogenesis, tricarboxylic acid cycle (TCA) cycle, purine metabolism, pyrimidine metabolism, fatty acid degradation, amino acid, and peptidoglycan biosynthesis], and transport hub (ATP-binding cassette transporters and protein export) (Figure 4B and Table S5).

As a result, the ribosome is the major group of significantly upregulated genes compared with other classified clusters (Figure 4). Most of the genes belonging to the ribosome cluster were upregulated (Table 1), in which 39 genes were significantly upregulated, accounting for 12.4% of all the DEGs. The ribosomal assembly related genes encoding 30S and 50S ribosomal subunit proteins (e.g., *rplO, rpsK, rplV, rplP, rpsD, rplR, rpsC, rpsE*, and *rplB*) were all upregulated in the recombinant pGUS-expressing *E. coli* (Table 1) and in *Pseudomonas aeruginosa* (Crabbé et al., 2011). The aminoacyl-transfer RNA (tRNA) biosynthesis were also reinforced under SMG (Table 2), in which the DEGs accounts for 2.9% of the total DEGs. The transcriptional levels of the DNA-directed RNA polymerase genes (including *rpoA, rpoB, rpoC*, and *rpoZ*) were also observed to be upregulated (Table 3). These upregulated ribosome-related genes and RNA polymerase genes may increase the rate of protein synthesis and contribute to the enhancement of the protein production under SMG environment.

**Table 1:** Transcription changes of ribosomal genes.

**Gene**	**Function descriptions**	**log_**2**_(SMG/NG)**	**–lg(*P*-value)**
*rpmE*	50S ribosomal subunit protein L31	17.46	0.40
*ykgM*	putative ribosomal protein	17.46	0.40
*rplO*	50S ribosomal subunit protein L15	2.09	145.07
*rpsK*	30S ribosomal subunit protein S11	2.01	83.80
*rplV*	50S ribosomal subunit protein L22	2.00	64.04
*rplP*	50S ribosomal subunit protein L16	1.94	55.21
*rpsD*	30S ribosomal subunit protein S4	1.87	202.58
*rplR*	50S ribosomal subunit protein L18	1.84	79.02
*rpmC*	50S ribosomal subunit protein L29	1.83	28.01
*rpsS*	30S ribosomal subunit protein S19	1.82	53.62
*rplQ*	50S ribosomal subunit protein L17	1.78	47.84
*rplW*	50S ribosomal subunit protein L23	1.72	42.12
*rpsC*	30S ribosomal subunit protein S3	1.69	113.34
*rpsE*	30S ribosomal subunit protein S5	1.69	112.30
*rplD*	50S ribosomal subunit protein L4	1.66	62.19
*rpmA*	50S ribosomal subunit protein L27	1.64	18.69
*rplT*	50S ribosomal subunit protein L20	1.61	47.36
*rplB*	50S ribosomal subunit protein L2	1.60	96.49
*rpsQ*	30S ribosomal subunit protein S17	1.52	32.19
*rplF*	50S ribosomal subunit protein L6	1.51	78.93
*rplC*	50S ribosomal subunit protein L3	1.50	54.34
*rplE*	50S ribosomal subunit protein L5	1.49	67.09
*rpsN*	30S ribosomal subunit protein S14	1.42	47.74
*rpmJ*	50S ribosomal subunit protein L36	1.42	34.72
*ykgO*	putative ribosomal protein	1.42	34.72
*rpsH*	30S ribosomal subunit protein S8	1.41	43.43
*rpmD*	50S ribosomal subunit protein L30	1.40	14.19
*rpsM*	30S ribosomal subunit protein S13	1.40	36.00
*rpmI*	50S ribosomal subunit protein L35	1.30	35.67
*rpsA*	30S ribosomal subunit protein S1	1.18	116.84
*rplJ*	50S ribosomal subunit protein L10	1.17	23.71
*rpsJ*	30S ribosomal subunit protein S10	1.07	19.14
*rplL*	50S ribosomal subunit protein L12	0.99	11.74
*rplX*	50S ribosomal subunit protein L24	0.94	12.37
*rplS*	50S ribosomal subunit protein L19	0.91	7.33
*rpmF*	50S ribosomal subunit protein L32	0.72	2.64
*rpmG*	50S ribosomal subunit protein L33	0.71	2.23
*rplN*	50S ribosomal subunit protein L14	0.69	11.25
*rplA*	50S ribosomal subunit protein L1	0.61	8.11
*rpsR*	30S ribosomal subunit protein S18	0.60	1.26
*rpmB*	50S ribosomal subunit protein L28	0.58	1.78
*rpsP*	30S ribosomal subunit protein S16	0.54	2.36
*rplI*	50S ribosomal subunit protein L9	0.52	1.25
*rpsO*	30S ribosomal subunit protein S15	0.48	4.68
*rplM*	50S ribosomal subunit protein L13	0.46	2.95
*rpsB*	30S ribosomal subunit protein S2	0.45	3.89
*rplU*	50S ribosomal subunit protein L21	0.36	1.27
*rplK*	50S ribosomal subunit protein L11	0.33	1.87
*rpsT*	30S ribosomal subunit protein S20	0.28	0.27
*rpsG*	30S ribosomal subunit protein S7	0.25	0.77
*rpsI*	30S ribosomal subunit protein S9	0.23	0.60
*rpsL*	30S ribosomal subunit protein S12	0.19	0.41
*rplY*	50S ribosomal subunit protein L25	0.16	0.18
*rpsU*	30S ribosomal subunit protein S21	−0.18	0.99
*rpsF*	30S ribosomal subunit protein S6	−0.53	8.39

**Table 2:** Transcription changes of aminoacyl-tRNA biogenesis genes.

**Gene**	**Function descriptions**	**log_**2**_** **(SMG/NG)**	**–lg(*P*-value)**
*cysS*	Phenylalanine–tRNA ligase subunit alpha	1.13	3.73
*ileS*	Isoleucine–tRNA ligase	1.12	44.21
*glyS*	Glycine–tRNA ligase subunit beta	0.92	7.04
*aspS*	Aspartate–tRNA ligase	0.92	6.23
*valS*	Valine–tRNA ligase	0.80	11.71
*proS*	Proline–tRNA ligase	0.76	3.58
*metG*	Methionine–tRNA ligase	0.75	3.92
*alaS*	Alanine–tRNA ligase/DNA-binding transcriptional repressor	0.65	12.77
*selA*	Selenocysteine synthase	0.64	0.34
*asnS*	Asparagine–tRNA ligase	0.56	2.41
*pheT*	Phenylalanine–tRNA ligase subunit beta	0.46	3.20
*lysS*	Lysine–tRNA ligase, constitutive	0.44	1.25
*lysU*	Lysine–tRNA ligase/Ap4A synthetase/Ap3A synthetase	0.44	1.25
*leuS*	Leucine–tRNA ligase	0.38	2.53
*argS*	Arginine–tRNA ligase	0.29	0.44
*glyQ*	Glycine–tRNA ligase subunit alpha	0.29	0.41
*gltX*	Glutamate–tRNA ligase	0.21	0.39
*thrS*	Threonine–tRNA ligase	0.21	1.08
*tyrS*	Tyrosine–tRNA ligase	0.05	0.05
*hisS*	Histidine–tRNA ligase	0.01	0.20
*serS*	Serine–tRNA ligase	0.00	0.25
*cysS*	Cysteine–tRNA ligase	0.00	0.14
*trpS*	Tryptophan–tRNA ligase	−0.20	0.81
*glnS*	Glutamine–tRNA ligase	−0.57	6.68
*fmt*	10-formyltetrahydrofolate:l-methionyl-tRNA(fMet) *N*-formyltransferase	−0.71	5.78

**Table 3:** Transcription changes of RNA polymerase genes.

**Gene**	**Function descriptions**	**log_**2**_(SMG/NG)**	**–lg(*P*-value)**
*rpoA*	RNA polymerase subunit alpha	1.82	270.07
*rpoB*	RNA polymerase subunit beta	2.03	200.53
*rpoC*	RNA polymerase subunit beta'	1.72	134.96
*rpoZ*	RNA polymerase subunit omega	0.59	0.69

The overall transcriptional level of glycolysis was upregulated (Table 4 and Figure S1), which could contribute to the enhanced uptake of carbon sources and the conversion of precursors to biomass. The aerotaxis receptor aer was upregulated by 2.99-fold (log_2_). The upregulation of gene *aer* indicated that SMG could guide cells toward oxygen and energy-generating niches. Most of oxidative phosphorylation genes (encoding NADH dehydrogenase, succinate dehydrogenase, cytochrome c oxidase, cytochrome bd complex, and ATPase) were upregulated (Table 5). These upregulated energy-generating genes may directly lead to high metabolic efficiency and high cell viability.

**Table 4:** Transcription changes of glycolysis genes.

**Gene**	**Function descriptions**	**log_**2**_** **(SMG/NG)**	**–lg(*P*-value)**
*chbF*	Monoacetylchitobiose-6-phosphate hydrolase	17.24	2.14
*glpX*	Fructose-1,6-bisphosphatase 2	16.02	0.57
*yggF*	Fructose 1,6-bisphosphatase	16.02	0.57
*fbaA*	Fructose-bisphosphate aldolase class II	15.23	0.22
*ascB*	6-phospho-beta-glucosidase	15.08	0.40
*bglA*	6-phospho-beta-glucosidase A	15.08	0.40
*bglB*	6-phospho-beta-glucosidase B	15.08	0.40
*eutG*	Putative alcohol dehydrogenase in ethanolamine utilization	14.72	0.22
*glk*	Glucokinase	2.22	1.56
*ascF*	Beta-glucoside specific PTS enzyme IIBC component	1.05	0.32
*frmA*	S-(hydroxymethyl)glutathione dehydrogenase	1.02	1.42
*aceF*	Pyruvate dehydrogenase, E2 subunit	0.92	20.53
*aldB*	Aldehyde dehydrogenase B	0.91	3.69
*yeaD*	Putative aldose 1-epimerase	0.85	1.58
*pfkA*	6-phosphofructokinase I	0.82	1.40
*pgi*	Glucose-6-phosphate isomerase	0.64	0.11
*yiaY*	L-threonine dehydrogenase	0.64	0.11
*ptsG*	Glucose-specific PTS enzyme IIBC component	0.61	0.64
*pykF*	Pyruvate kinase I	0.54	1.59
*pykA*	Pyruvate kinase II	0.54	1.59
*aceE*	Pyruvate dehydrogenase E1 component	0.51	9.80
*ydbK*	Putative pyruvate-flavodoxin oxidoreductase	0.49	1.13
*acs*	Acetyl-CoA synthetase (AMP-forming)	0.36	5.54
*eno*	Enolase	0.36	1.31
*gpmA*	2,3-bisphosphoglycerate-dependent phosphoglycerate mutase	0.34	0.31
*agp*	Glucose-1-phosphatase	0.19	0.63
*lpd*	Lipoamide dehydrogenase	0.11	0.30
*adhP*	Ethanol dehydrogenase/alcohol dehydrogenase	0.09	0.01
*pck*	Phosphoenolpyruvate carboxykinase (ATP)	0.04	0.21
*pgm*	Phosphoglucomutase	−0.09	0.73
*pgk*	Phosphoglycerate kinase	−0.13	1.11
*gapA*	Glyceraldehyde-3-phosphate dehydrogenase A	−0.19	5.79
*crr*	Enzyme IIA(Glc)	−0.20	1.28
*fbp*	Fructose-1,6-bisphosphatase 1	−0.32	2.98
*adhE*	Aldehyde-alcohol dehydrogenase	−0.32	4.64
*tpiA*	Triose-phosphate isomerase	−0.36	1.95
*galM*	Galactose-1-epimerase	−0.53	0.30

**Table 5:** Transcription changes of oxidative phosphorylation genes.

**Gene**	**Function descriptions**	**log_**2**_** **(SMG/NG)**	**–lg(*P*-value)**
*nuoJ*	NADH:quinone oxidoreductase subunit J	1.20	2.54
*frdC*	Fumarate reductase membrane protein FrdC	1.19	0.92
*cydB*	Cytochrome bd-I ubiquinol oxidase subunit II	1.13	0.82
*appB*	Cytochrome bd-II ubiquinol oxidase subunit II	1.13	0.82
*nuoI*	NADH:quinone oxidoreductase subunit I	1.03	2.87
*nuoM*	NADH:quinone oxidoreductase subunit M	1.01	3.00
*nuoL*	NADH:quinone oxidoreductase subunit L	0.84	3.06
*nuoK*	NADH:quinone oxidoreductase subunit K	0.79	0.27
*nuoN*	NADH:quinone oxidoreductase subunit N	0.71	1.81
*atpA*	ATP synthase F1 complex subunit alpha	0.66	7.57
*atpH*	ATP synthase F1 complex subunit delta	0.64	1.98
*nuoF*	NADH:quinone oxidoreductase subunit F	0.60	2.95
*atpC*	ATP synthase F1 complex subunit epsilon	0.57	1.67
*atpB*	ATP synthase Fo complex subunit a	0.54	2.19
*nuoC*	NADH:quinone oxidoreductase subunit CD	0.52	3.85
*frdB*	Fumarate reductase iron-sulfur protein	0.49	0.78
*atpG*	ATP synthase F1 complex subunit gamma	0.48	1.83
*ndh*	NADH:quinone oxidoreductase II	0.47	0.85
*atpE*	ATP synthase Fo complex subunit c	0.38	0.36
*atpF*	ATP synthase Fo complex subunit b	0.36	1.37
*nuoG*	NADH:quinone oxidoreductase subunit G	0.36	2.30
*atpD*	ATP synthase F1 complex subunit beta	0.32	1.25
*sdhA*	Succinate:quinone oxidoreductase, FAD binding protein	0.31	5.46
*sdhB*	Succinate:quinone oxidoreductase, iron-sulfur cluster binding protein	0.30	2.10
*nuoE*	NADH:quinone oxidoreductase subunit E	0.20	0.33
*cyoB*	Cytochrome bo3 ubiquinol oxidase subunit 1	0.19	1.46
*frdA*	Fumarate reductase flavoprotein subunit	0.17	0.29
*sdhD*	Succinate:quinone oxidoreductase, membrane protein	0.04	0.08
*ppa*	Inorganic pyrophosphatase	0.01	0.16
*nuoH*	NADH:quinone oxidoreductase subunit H	−0.12	0.44
*cyoD*	Cytochrome bo3 ubiquinol oxidase subunit 4	−0.15	0.78
*cyoA*	Cytochrome bo3 ubiquinol oxidase subunit 2	−0.18	4.03
*cydA*	Cytochrome bd-I ubiquinol oxidase subunit I	−0.21	0.26
*appC*	Cytochrome bd-II ubiquinol oxidase subunit I	−0.21	0.26
*cyoC*	Cytochrome bo3 ubiquinol oxidase subunit 3	−0.22	2.10
*nuoB*	NADH:quinone oxidoreductase subunit B	−0.23	0.97
*sdhC*	Succinate:quinone oxidoreductase, membrane protein	−0.39	1.48
*nuoA*	NADH:quinone oxidoreductase subunit A	−0.54	1.94
*ppk*	Polyphosphate kinase	−0.81	6.17
*frdD*	Fumarate reductase membrane protein	−1.48	1.94

Interestingly, the genes about cysteine synthesis, cysW and cysH, were both substantially upregulated [increased by 17.93- and 17.58-fold (log2), respectively] (Figure 4A). Thus, cysteine, acting as a building unit for protein translation and involves in redox homeostasis, may contribute to higher recombinant protein production responding to SMG, while the mechanism is still unknown and needs to be further studied in future.

Protein folding is an outstanding feature issuing in efficient metabolism conversion under SMG. kdpE is a transcription factor for potassium homeostasis. In this study, *kdpE* was significantly upregulated by 17.85-fold (log_2_) under SMG (Figure 4A). Chaperone groEL is just one of the K^+^-activated type I enzymes. Therefore, it was suggested that SMG could provide a better environment for improving the activities of chaperones to reduce protein aggregation upon environmental stress. However, the transcriptional level of some chaperone genes did not show significant upregulation under SMG, such as *dnaJ, groEL, cpxP*, and *ppiD* (Tables S2–S4), which was also observed in previous studies (Tucker et al., 2007; Wilson et al., 2007). Meanwhile, *secY* encoding a transmembrane transporter was significantly upregulated (Figure 4A), suggesting that the activity of protein export was strengthened due to the high protein production.

## Proteomic Analysis Revealed the Mechanism of Enhanced Protein Expression Under SMG

Under SMG condition, there might be differential expression clusters of proteins to cope with the enhanced recombinant protein production which can prevent protein misfolding and protein aggregation. In proteomics investigation, the number of the DEPs are identified as 69 (without IPTG induction) and 199 (with IPTG induction) under SMG, respectively, compared with NG. Without IPTG induction, 32 proteins were significantly upregulated and only 1 protein (rpsS) was drastically downregulated (Table S6). With the induction, 181 proteins were upregulated and 6 proteins were downregulated (Table S8). The large amount of upregulated proteins reflected that SMG not only improved the production of heterogenous proteins but also increased the expression of most of endogenous proteins. The DEPs were classified into three hubs: metabolism hub (TCA cycle, glycolysis/gluconeogenesis, fatty acid biosynthesis, amino acid metabolism, pyrimidine/purine metabolism), translation hub (RNA polymerase, ribosome), and folding/transport hub (chaperone, protein export) (Tables S7, S9). Similar to the result of transcriptome, the differential pathways from proteome still focused on carbon metabolism, translation, and protein transport. Chaperone was discovered differentially expressed at protein level, which was not found in the transcriptional level.

**Figure 5:**
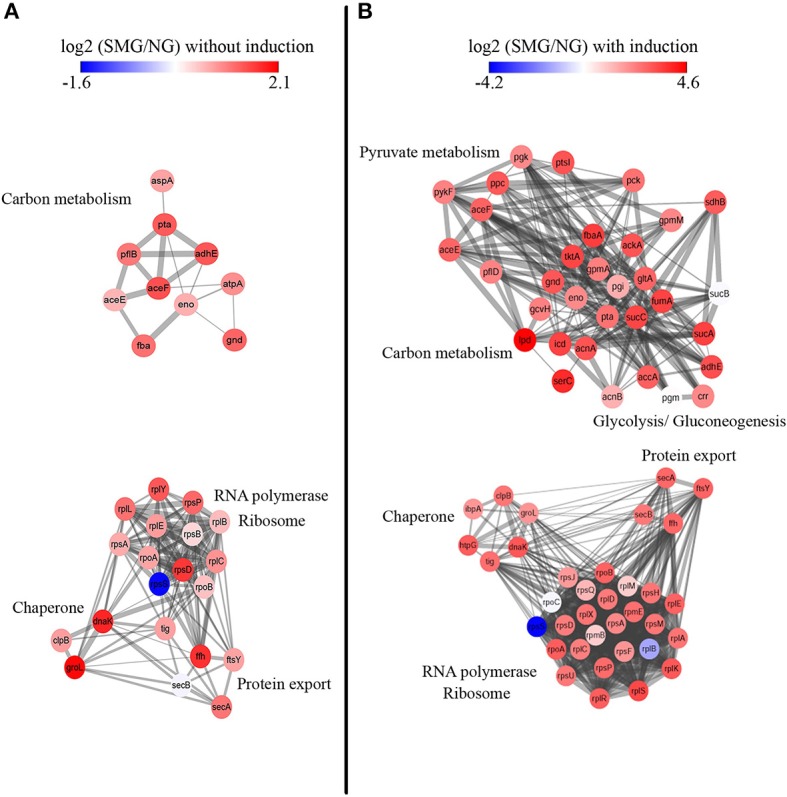
Protein–protein interaction network analysis. STRING clusters represent proteins involved in carbon metabolism and RNA polymerase, ribosome, chaperone, and protein export. Proteins are colored either in red (representing upregulation) or in blue (representing downregulation) according to their differential expression levels. Left panel **(A)** is the proteins without IPTG induction, and right panel **(B)** is the proteins with IPTG induction.

Protein–protein interactions were analyzed for deeper understanding (Figure 5). Without IPTG induction, chaperone proteins groEL (Hsp60), dnaK (Hsp70), and clpB (Hsp100) are mostly upregulated under SMG comparing to NG (log_2_ change fold was 2.03, 1.79, and 0.8, respectively) (Figure 5A). These proteins functioning as folding modulators are associated with inclusion body prevention. With the induction, we found that chaperones such as dnaK, groEL, ibpA, clpB, and htpG were all upregulated under SMG (log_2_ change fold was 3.02, 1.93, 1.88, 2.49, and 2.92, respectively) (Figure 5B). These upregulated DEPs suggested that SMG environment could enhance the translation and expression of chaperones to provide the suitable environment for correct protein folding.

It was found that proteins involving in Sec-dependent pathway also had differential expression levels under SMG. The upregulation of secA, secB, ffh, and ftsY showed that protein export is also an important step for high-efficient recombinant protein production (Figure 5).

In addition, a panel of enzymes involved in carbon metabolism was upregulated. For example, the expression of gltA (citrate synthase, a gatekeeper gene to TCA cycle) was upregulated by 2.81-fold (log_2_), which could improve energy metabolism and cell growth.

## Discussion

One of the obstacles for obtaining large amounts of recombinant proteins in *E. coli* is the inclusion bodies (De Marco, 2009). It is known that altering the growth conditions can affect soluble protein expression level by varying the folding environments of the recombinant protein, such as initial culture density, temperature, and duration of the expression stage (San-Miguel et al., 2013). Besides, the growth and induction of cells under heat-shock, osmotic stress, and osmole supplementation conditions have been shown to enhance solubility of some recombinant proteins (Harrison and Bagajewicz, 2015). However, there is still a limitation for further improvement of the protein production. In this study, SMG can be regarded as a special environment due to the variation of gravity, mass transfer, and nutrient supply for cell to respond and thus have physiochemical changes. Several studies have found that SMG enhanced the production of recombinant proteins (Navran, 2007; Xiang et al., 2010; Huangfu et al., 2016). According to the omics analysis in this study, the crucial upregulated clusters under SMG are the groups of ribosomes/RNA polymerase genes, which directly contributed to the high-efficient protein synthetic ratio. Transcriptomic data of our study were compared with the previous studies about *P. aeruginosa* (Crabbé et al., 2011), *E. coli* K12 (Vukanti et al., 2008), and *Salmonella typhimurium* (Wilson et al., 2007). Most of the upregulated genes in the ribosome and RNA polymerase were in line with these previous studies. We have overexpressed certain ribosome genes which were significantly upregulated in this omics study (data not shown). Unfortunately, this approach resulted in unremarkable improvement of protein production because the overexpression of a few genes cannot increase the overall ribosome/RNA polymerase level, which was regulated by dozens of genes. To monitor ribosome dynamics or to map ribosome profiling might be helpful to improve protein production in the future. Chaperones demonstrated more of upregulation at proteomic level than that of the transcriptomic level under SMG. The overexpression of chaperones may be a result of the increased ribosome activity (ability to produce proteins). Thus, the highly expressed chaperones could reduce protein aggregation resulting in further improved recombinant protein quality. Previous studies suggested that folding modulators including dnaK, clpB, and groL were overexpressed as the culture temperature increases (Hoffmann and Rinas, 2010). Since the overexpression of heterologous protein was regarded as an intracellular stress which may aggravate protein misfolding, the upregulated chaperones can be helpful to prevent the formation of inclusion bodies (Baneyx and Mujacic, 2004). Sec system is the main pathway for protein secretion, in which secY and secA exert important roles (Allen et al., 2016). Our result showed that secY and secA were significantly upregulated at both transcriptomic and proteomic levels, respectively, which implicated the activity of protein export was substantially increased under SMG.

**Figure 6:**
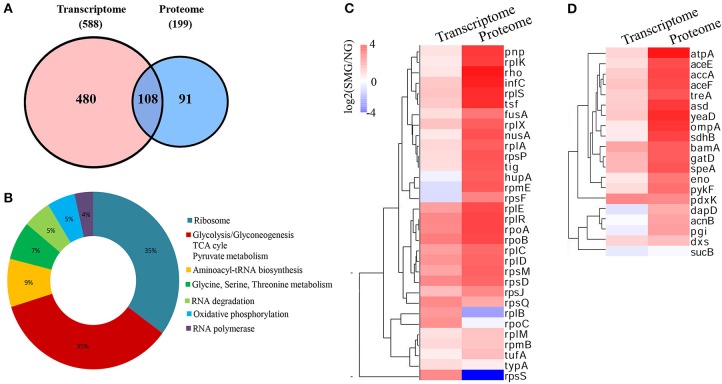
Comprehensive comparison between transcriptomics and proteomics. **(A)** The intersection between transcriptome and proteome. **(B)** The clusters in the intersection. **(C)** Ribosome cluster in the intersection. **(D)** Carbon metabolism cluster in the intersection.

As known, because mRNAs would go through complicated translational regulations, there are always inconsistent genes/proteins between the transcriptomics and proteomics data, which can reflect cells undergoing different states. We analyzed both the consistent and inconsistent genes/proteins between the transcriptomics and the proteomics. Between the DEGs and DEPs, 108 genes/proteins were at the intersection in both the transcriptomics and proteomics profiling (Figure 6A). Ribosome and carbon metabolism were the two biggest clusters of the intersection, both of which accounted for 35% of the overlapped genes/proteins, followed by aminoacyl-tRNA biosynthesis (9%), glycine/serine/threonine metabolism (7%), RNA degradation (5%), oxidative phosphorylation (5%), and RNA polymerase (4%) (Figure 6B). Most of the ribosome and glycolytic enzymes are found more upregulated in the protein level than that in the transcription level under SMG, such as *rplA, eno, pykF*, etc. (Figures 6C,D). For example, *tig* (encoding the Trigger factor) is upregulated by 0.5-fold (log_2_) in transcriptomic level and 2.59-fold (log_2_) in proteomic level. Meanwhile, a few genes were not consistent between the transcriptomics and the proteomics. For example, *rplB* and *rpsS* were upregulated at transcriptional level, while their encoded proteins were downregulated under SMG (Figure 6C). This suggested that the SMG environment could influence the translation process of some specific mRNA in an unknown manner, which is an important subject and needs to be studied in depth in the future.

## Data Availability Statement

All datasets generated for this study are included in the article/Supplemental Material.

## Author Contributions

JH, HK, KX, and XN carried out the experiments and drafted the manuscript. JH, JL, and CL participated in experimental design and supported the experiments. LQ, JL, and CL conceived and coordinated the study and drafted the manuscript. All authors read and approved the final manuscript.

### Conflict of Interest

The authors declare that the research was conducted in the absence of any commercial or financial relationships that could be construed as a potential conflict of interest.

## References

[B1] Allen W. J. Corey R. A. Oatley P. Sessions R. B. Baldwin S. A. Radford S. E. (2016). Two-way communication between SecY and SecA suggests a Brownian ratchet mechanism for protein translocation. Elife 16:e15598 10.7554/eLife.15598.031 PMC4907695 27183269

[B2] Baker P. W. Meyer M. L. Leff L. G. (2004). *Escherichia coli* growth under modeled reduced gravity. Microgravity Sci. Technol. 15, 39–44. 10.1007/BF02870967 15768486

[B3] Baneyx F. Mujacic M. (2004). Recombinant protein folding and misfolding in *Escherichia coli*. Nat. Biotechnol. 22, 1399–1408. 10.1038/nbt1029 15529165

[B4] Brown R. B. Klaus D. Todd P. (2002). Effects of space flight, clinorotation, and centrifugation on the substrate utilization efficiency of *E. coli*. Microgravity Sci. Technol. 13:24. 10.1007/BF02881678 12521048

[B5] Clerico E. M. Tilitsky J. M. Meng W. Gierasch L. M. (2015). How hsp70 molecular machines interact with their substrates to mediate diverse physiological functions. J. Mol. Biol. 427, 1575–1588. 10.1016/j.jmb.2015.02.004 25683596 PMC4440321

[B6] Crabbé A. Schurr M. J. Monsieurs P. Morici L. Schurr J. Wilson J. W.. (2011). Transcriptional and proteomic responses of *Pseudomonas aeruginosa* PAO1 to spaceflight conditions involve Hfq regulation and reveal a role for oxygen. Appl. Environ. Microbiol. 77, 1221–1230. 10.1128/AEM.01582-10 21169425 PMC3067220

[B7] De Marco A. (2009). Strategies for successful recombinant expression of disulfide bond-dependent proteins in *Escherichia coli*. Microbial Cell Factories 8:26. 10.1186/1475-2859-8-26 19442264 PMC2689190

[B8] Feng S. Li C. Xu X. Wang X. (2006). Screening strains for directed biosynthesis of β-d-mono-glucuronide-glycyrrhizin and kinetics of enzyme production. J. Mol. Catal. B Enzymat. 43, 63–67. 10.1016/j.molcatb.2006.06.016

[B9] Frydman J. Hartl F. U. (1996). Principles of chaperone-assisted protein folding: differences between *in vitro* and *in vivo* mechanisms. Science 272, 1497–1502. 10.1126/science.272.5267.1497 8633246

[B10] Gao Q. Fang A. Pierson D. Mishra S. Demain A. (2001). Shear stress enhances microcin B17 production in a rotating wall bioreactor, but ethanol stress does not. Appl. Microbiol. Biotechnol. 56, 384–387. 10.1007/s002530100610 11549006

[B11] Harrison R. G. Bagajewicz M. J. (2015). Predicting the solubility of recombinant proteins in *Escherichia coli*. Methods Mol. Biol. 1258, 403–408. 10.1007/978-1-4939-2205-5_23 25447878

[B12] Hartl F. U. Hayer-Hartl M. (2009). Converging concepts of protein folding *in vitro* and *in vivo*. Nat. Struct. Mol. Biol. 16, 574–581. 10.1038/nsmb.1591 19491934

[B13] Hayer-Hartl M. Bracher A. Hartl F. U. (2016). The GroEL–GroES chaperonin machine: a nano-cage for protein folding. Trends Biochem. Sci. 41, 62–76. 10.1016/j.tibs.2015.07.009 26422689

[B14] Hoffmann F. Rinas U. (2004). Roles of heat-shock chaperones in the production of recombinant proteins in *Escherichia coli*. Adv. Biochem. Eng. Biotechnol. 89, 143–161. 10.1007/b93996 15217158

[B15] Hoffmann F. Rinas U. (2010). Kinetics of heat-shock response and inclusion body formation during temperature-induced production of basic fibroblast growth factor in high-cell-density cultures of recombinant *Escherichia coli*. Biotechnol. Prog. 16, 1000–1007. 10.1021/bp0000959 11101327

[B16] Huangfu J. Xu Y. Li C. Li J. (2016). Overexpressing target helper genes enhances secretion and glycosylation of recombinant proteins in *Pichia pastoris* under simulated microgravity. J. Ind. Microbiol. Biotechnol. 43, 1429–1439. 10.1007/s10295-016-1817-8 27535143

[B17] Jamal A. Ko K. Kim H.-S. Choo Y.-K. Joung H. Ko K. (2009). Role of genetic factors and environmental conditions in recombinant protein production for molecular farming. Biotechnol. Adv. 27, 914–923. 10.1016/j.biotechadv.2009.07.004 19698776

[B18] Kaltschmidt E. Wittmann H. G. N. (1970). Ribosomal proteins, XII. Number of proteins in small and large ribosomal subunits of *Escherichia coli* as determined by two-dimensional gel electrophoresis. Proc. Natl. Acad. Sci. U.S.A. 67, 1276–1282. 10.1073/pnas.67.3.1276 4922286 PMC283348

[B19] Navran S. (2007). Rotary bioreactor for recombinant protein production. Cell Technol. Cell Prod. 567–569. 10.1007/978-1-4020-5476-1_98

[B20] Nickerson C. A. Ott C. M. Wilson J. W. Ramamurthy R. Pierson D. L. (2004). Microbial responses to microgravity and other low-shear environments. Microbiol. Mol. Biol. Rev. 68, 345–361. 10.1128/MMBR.68.2.345-361.2004 15187188 PMC419922

[B21] Ogle J. M. Brodersen D. E. Clemons W. M. Tarry M. J. Carter A. P. Ramakrishnan V. (2001). Recognition of cognate transfer RNA by the 30S ribosomal subunit. Science 292, 897–902. 10.1126/science.1060612 11340196

[B22] Rosenzweig J. A. Abogunde O. Thomas K. Lawal A. Nguyen Y.-U. Sodipe A.. (2010). Spaceflight and modeled microgravity effects on microbial growth and virulence. Appl. Microbiol. Biotechnol. 85, 885–891. 10.1007/s00253-009-2237-8 19847423 PMC2804794

[B23] San-Miguel T. Pérez-Bermúdez P. Gavidia I. (2013). Production of soluble eukaryotic recombinant proteins in *E. coli* is favoured in early log-phase cultures induced at low temperature. Springerplus 2:89. 10.1186/2193-1801-2-89 23525091 PMC3602615

[B24] Shi Y. Yang X. Garg N. van der Donk W. A. (2011). Production of lantipeptides in *Escherichia coli*. J. Am. Chem. Soc. 133, 2338–2341. 10.1021/ja109044r 21114289 PMC3044485

[B25] Takyar S. Hickerson R. P. Noller H. F. (2005). mRNA helicase activity of the ribosome. Cell 120, 49–58. 10.1016/j.cell.2004.11.042 15652481

[B26] Tucker D. L. Ott C. M. Huff S. Fofanov Y. Pierson D. L. Willson R. C.. (2007). Characterization of *Escherichia coli* MG1655 grown in a low-shear modeled microgravity environment. BMC Microbiol. 7:15. 10.1186/1471-2180-7-15 17343762 PMC1852313

[B27] Vukanti R. Mintz E. Leff L. (2008). Changes in gene expression of *E. coli* under conditions of modeled reduced gravity. Microgravity Sci. Technol. 20:41 10.1007/s12217-008-9012-9

[B28] Wilson J. Ott C. Zu Bentrup K. H. Ramamurthy R. Quick L. Porwollik S.. (2007). Space flight alters bacterial gene expression and virulence and reveals a role for global regulator Hfq. Proc. Natl. Acad. Sci. U.S.A. 104, 16299–16304. 10.1073/pnas.0707155104 17901201 PMC2042201

[B29] Wilson J. W. Ott C. M. Ramamurthy R. Porwollik S. McClelland M. Pierson D. L.. (2002). Low-shear modeled microgravity alters the *Salmonella enterica* serovar Typhimurium stress response in an RpoS-independent manner. Appl. Environ. Microbiol. 68, 5408–5416. 10.1128/AEM.68.11.5408-5416.2002 12406731 PMC129924

[B30] Xiang L. Qi F. Dai D. Li C. Jiang Y. (2010). Simulated microgravity affects growth of *Escherichia coli* and recombinant β-D-glucuronidase production. Appl. Biochem. Biotechnol. 162, 654–661. 10.1007/s12010-009-8836-0 19921492

