# Supplementary material for: Omics Analysis Reveals the Mechanism of Enhanced Recombinant Protein Production Under Simulated Microgravity
Source: Front Bioeng Biotechnol. 2020 Feb 20;8:30. doi: 10.3389/fbioe.2020.00030 (PMC7044180; doi:10.3389/fbioe.2020.00030)
Supplement: Supplementary file 1 [file Data_Sheet_1.docx]

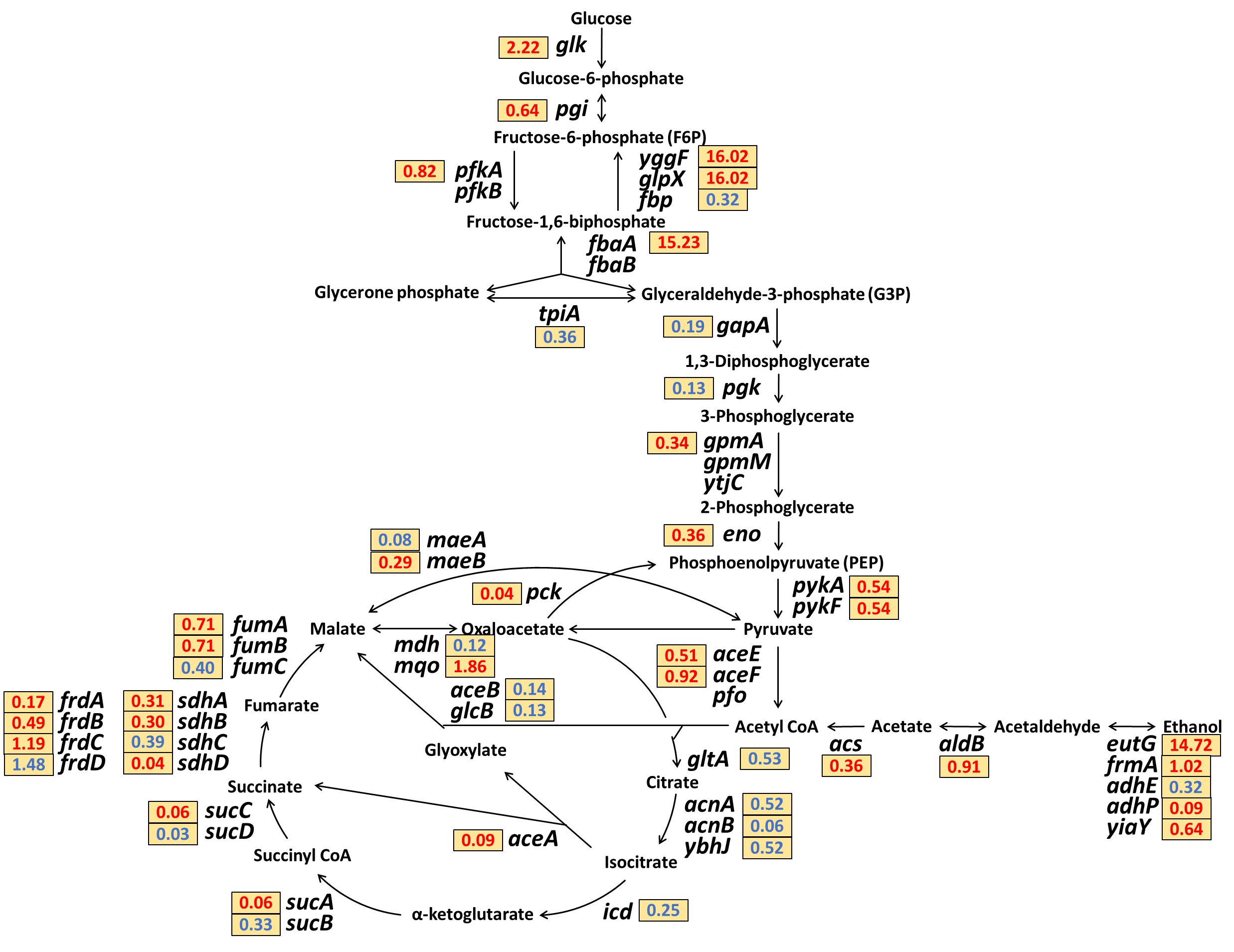


**Figure S1** Transcriptional change of center carbon metabolism of recombinant protein production *E. coli* under SMG compared with NG. Red characters stand for log_2_(SMG/NG) of up-regulated genes and blue characters stand for log_2_(SMG/NG) of down-regulated genes.
